# Distinguishing Features of ILC2s During Type 2 Immune Responses

**DOI:** 10.1002/eji.70151

**Published:** 2026-02-12

**Authors:** Manuel O. Jakob, Patrycja M. Forster, Christoph S. N. Klose

**Affiliations:** ^1^ Charité – Universitätsmedizin Berlin, Corporate Member of Freie Universität Berlin and Humboldt‐Universität zu Berlin, Department of Microbiology Infectious Diseases and Immunology Berlin Germany; ^2^ Department of Visceral Surgery and Medicine Inselspital, Bern University Hospital University of Bern Bern Switzerland; ^3^ Cluster of Excellence Immunoprecept Charité ‐ Universitätsmedizin Berlin Berlin Germany

**Keywords:** Innate immunity, innate lymphoid cells, mucosal immunology, type 2 immunity

## Abstract

Innate lymphoid cells (ILCs) are innate immune cells populating many tissues early in ontogeny. ILCs and T cells share basic transcriptional programs, immune modules, and effector functions. This article focuses on ILC2s, a subset capable of producing an array of type 2 cytokines similar to T helper 2 (Th2) cells. While there is a striking overlap in effector functions between ILC2s and Th2 cells, we discuss the distinguishing features and nonredundant functions of ILC2s in homeostasis and during inflammation. These include the regulation of their effector functions, the kinetics of their response, and their spatial distribution. ILC2s interact with different cell types and maintain crosstalk with nonimmune cells, such as epithelial cells, stromal cells, and neurons. We review how neurons and epithelial cells trigger ILC2 responses and depend on ILC2‐derived cytokines, leading to the concept that expulsion of many gut‐dwelling nematodes is initiated and executed by the intestinal epithelium but strictly requires the type 2 immune response orchestrated by ILC2s.

## Graphical abstract

Group 2 innate lymphoid cells (ILC2s) are tissue‐resident cells regulated by diverse signals often emanating from surrounding nonhematopoietic cells at barrier surfaces. Their effector cytokines determine tissue homeostasis at steady state and during type 2 immunity in allergies or worm infections. Redundant and unique functions of ILC2s and T cells are assessed in this context.

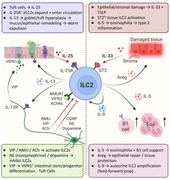

**Abbreviations**
AHR: aryl hydrocarbon receptor
Areg: amphiregulin
CGRP: calcitonin gene‐related peptide
CMV: cytomegalovirus
CystLT: cysteinyl leukotriene
GATA‐3: GATA binding protein 3
GM‐CSF: granulocyte‐macrophage colony‐stimulating factor
HSCT: hematopoietic stem cell transplantation
IL: interleukin
IL‐7R: interleukin 7 receptor
ILC: innate lymphoid cell
JAK: Janus tyrosine kinases
LIF: leukemia inhibitory factor
LTi cells: lymphoid tissue inducer cells
NK cells: natural killer cells
NKG2D: natural killer group 2 member D
NMUR1: neuromedin U receptor 1
RAR: retinoic acid receptors
RORγt: retinoic acid receptor‐related orphan receptor gamma t
SCID: severe combined immunodeficiency
T‐bet: T‐box transcription factor TBX21
TGF‐β: transforming growth factor‐β
TSLP: thymic stromal lymphopoietin
VIPR: vasoactive intestinal peptide receptor

## 1 Introduction—ILC2s in Their Microenvironment in Tissue Niches

Research over the last 15 years in mice and humans has exposed distinct innate lymphoid cell (ILC) lineages with lineage‐specifying transcription factors and effector programs mirroring the functional diversity of T cells [1, 2]. Based on genetic and functional data, cytotoxic ILCs lacking the interleukin 7 receptor (IL‐7R, CD127), represented only by classical Eomes^+^ natural killer (NK) cells, and IL‐7R^+^ helper‐like ILCs are distinguished [3, 4, 5]. However, specialized NK cell subsets, such as thymic NK cells expressing CD127, have been described [6], and CD127 expression in ILC2s can be downregulated upon activation [7, 8]. NK cells were already described in the 1970s since they are represented in secondary lymphoid organs and express CD62L to leave the blood through high endothelial venules and enter the lymph nodes, similar to T cells [9]. In contrast, IL‐7R^+^ ILC subsets, which include Group 1 ILCs (ILC1s) (T‐bet) [4, 5, 10], ILC2s (GATA‐3) [11, 12, 13, 14, 15, 16], and ILC3s (RORγt) [17, 18, 19, 20, 21, 22, 23] were mainly described as immune effector populations from 2009 onwards and were barely found in the blood and secondary lymphoid organs, with the exception of mucosal‐associated lymph structures. Murine ILCs establish tissue‐residency, with the capacity to exit tissues under certain conditions, whereas human ILCs are present in the blood circulation [7, 16, 24]. Recently, this model has been extended as NK cells establish tissue residency after infection, whereas an activated subset of ILC2s, termed inflammatory ILC2s (iILC2s), elicited during worm infection can migrate into blood, lymphs, and distant tissues/organs as well [25, 26, 27].

**FIGURE 1:**
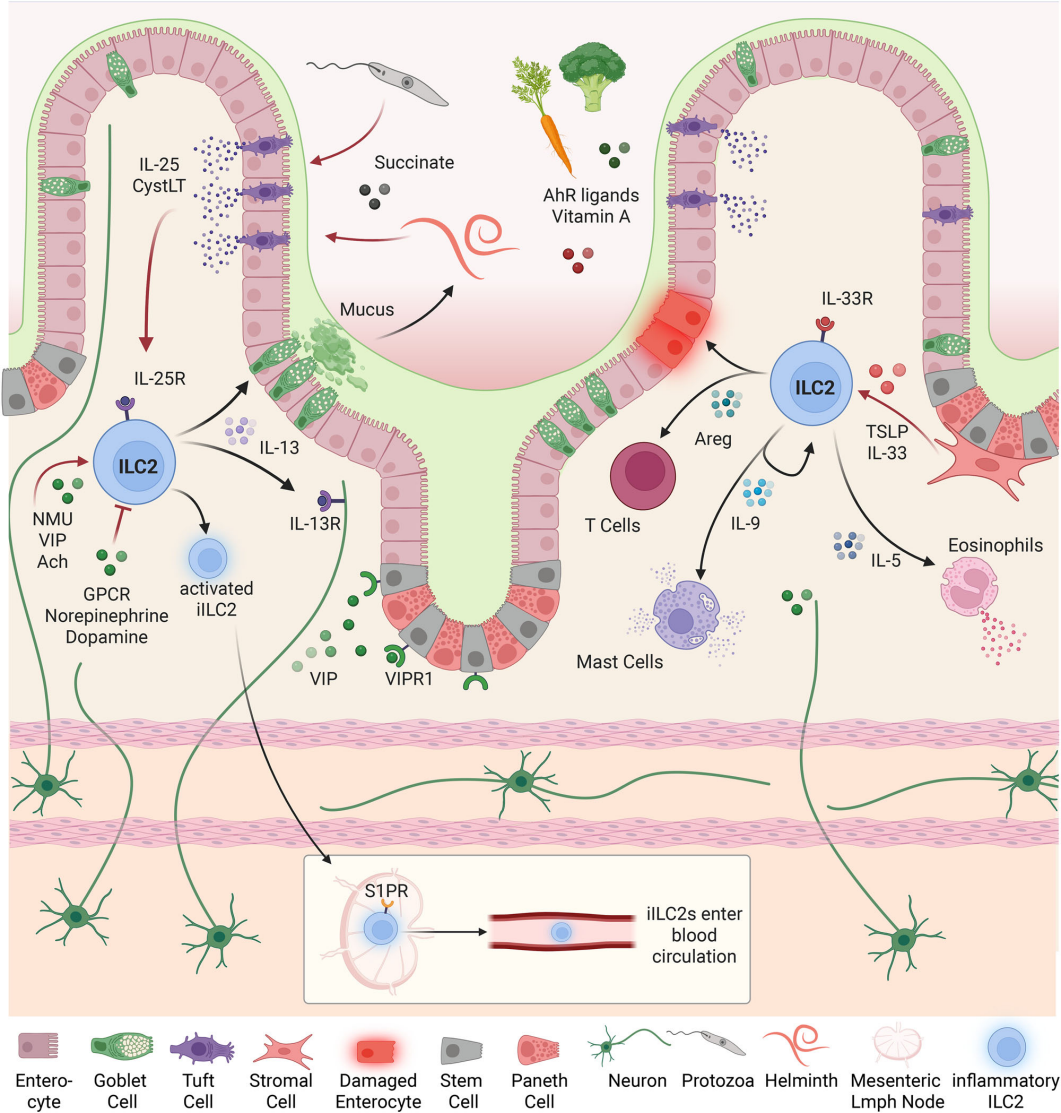
**ILC2s as central regulators of type 2 immunity at mucosal barriers**. ILC2 activation is regulated by different factors, as exemplified in the intestinal mucosa, one of several mucosal barrier surfaces. The tuft cell‐ILC2 circuit mediated by IL‐25 (depicted on the left) induces IL‐13 secretion, promoting worm resistance and migration of inflammatory ILC2s (iILC2s). On the right, the IL‐33 pathway, which is linked to IL‐5 secretion and eosinophilia, is depicted. While either IL‐25 or IL‐33 may predominate in certain settings, substantial overlap between these pathways is very common, and in many settings, both cytokines are required. The precise mechanisms governing their integration and regulation remain to be fully elucidated. GPCR, G‐protein coupled receptors; NMU, neuromedin U; VIP, vasoactive intestinal peptide; Ach, acetylcholine; TSLP, thymic stromal lymphopoietin; CystLT, cysteinyl leukotriene. *Source*: Created in BioRender. Jakob, M. (2026) https://BioRender.com/dsd4yhj.

ILC2 are enriched at mucosal barriers of the gastrointestinal and respiratory tract but can be found in virtually any tissue, such as fat, mesenteric lymph nodes, liver, and pancreas [1, 2]. ILC2s maintain multiple interactions with nonhematopoietic cell types in their environment, complementing the crosstalk with other immune cells. Interaction partners of ILC2s within tissues include epithelial cells, stromal cells, neurons, adipocytes, and muscle cells. In addition, ILC2s detect soluble factors such as metabolites, hormones, cytokines, and chemokines [28, 29, 30]. Thus, epithelial and stromal cells have a decisive role in the initiation of effector functions of ILC2s. The dominant cytokine in the intestine is IL‐25 through its tonic release from tuft cells, which drives the presence of IL‐25R^+^ ILC2s that reside primarily in the intestinal tissue at homeostasis [31, 32, 33]. Other tissues, such as the lung, where IL‐33 is the prevalent cytokine expressed by stromal cells and epithelial cells, contain mostly IL‐33R^+^ (ST2^high^) ILC2s [7, 34, 35] (Figure 1). While the phenotypic subdivision of ILC2s into IL‐25R^+^ and IL‐33R^+^ subsets in mice has proven useful, they are expected to represent distinct activation stages rather than separate lineages.

### 1.1 Initiation, Amplification, and Regulation of ILC2 Responses

The capabilities of ILC2s to directly sense pathogens, for example via pattern recognition receptors, are very limited in mice. However, human ILC2s have been shown to express several Toll‐like receptors, enabling direct responsiveness to microbial cues and promoting type 2 cytokine production [36]. In mice and humans, a broad array of mainly soluble factors regulates ILC2 activation in tissues [1, 2]. Upon stimulation (e.g., with IL‐25, IL‐33, and others), ILC2s acquire an activated phenotype, giving rise to iILC2s, an ILC2‐subset characterized by high KLRG1 expression, with low ST2 and CD25 levels, and the capacity to migrate between organs. Interestingly, the alarmins IL‐25, IL‐33, and thymic stromal lymphopoietin (TSLP) are released upon sensing danger signals or tissue damage and are primarily produced by nonhematopoietic cells, with immune cells contributing relatively little despite their role as sensors of tissue perturbation [37, 38, 39, 40]. This raises the question of how alarmin release is specifically regulated. In the intestine, Tuft cells are a rare type of secretory epithelial cells and the main source of the cytokine IL‐25 [31, 32, 33]. Tuft cells are chemosensory sentinel cells that detect succinate produced by helminth or protozoan parasites, leading to the release of IL‐25 and also of the inflammatory mediator cysteinyl leukotriene (CystLT) [41, 42]. Both molecules are sensed by the corresponding receptors IL‐25R and CystLTR expressed by ILC2s, playing a pivotal role in the initiation and maintenance of ILC2 responses. Tuft cells are crucial to trigger ILC2 activation via IL‐25 following helminth infection [31, 32, 33]. In addition, the inflammatory mediators, prostaglandin D2 (primarily produced by mast cells), activate ILC2s, whereas Prostaglandin E2 (generated by epithelial cells, fibroblasts, and macrophages) and lipoxin A4 (produced by eosinophils and airway epithelial cells) inhibit ILC2s [43, 44]. IL‐33 and TSLP are required for ILC2 activation [13, 14, 45, 46, 47], and are predominantly expressed by stromal cells and epithelial cells, and to a lesser extent by immune cells [37, 38, 39, 48, 49, 50, 51, 52, 53]. These cytokines are released in response to structural cell activation or damage, such as epithelial injury caused by nematode invasion or active cytokine release following allergen exposure [54, 55, 56]. Nevertheless, further investigation is required to dissect the relative contribution of these cellular sources across specific cell types and inflammatory contexts.

Additional cytokines have a pivotal role in the regulation of ILC2 responses. Among other cell types, IL‐9 is produced by ILC2s themselves following activation and, therefore, seems to amplify ILC2 responses in an auto‐ or paracrine manner [57, 58]. Some evidence suggests that IL‐4 can directly stimulate ILC2s via the cognate receptor IL‐4ra [59]. IL‐7 is strictly required for ILC2 development and homeostasis and can also support ILC2 activation [13, 60]. TGF‐β regulates IL‐33R expression during development but also boosts ILC2 cytokine production during allergic lung inflammation [61]. In contrast, cytokines promoting type 1 immune responses, such as IL‐27 and type I/II interferons, dampen type 2 immune responses via inhibition of ILC2s, which express the corresponding receptors [62, 63, 64].

Complementing cytokine‐mediated regulation, neuronal factors strongly regulate ILC2 function and activation. These include neuropeptides and neurotransmitters that engage cognate receptors expressed by ILC2s. ILC2s express a variety of receptors for sensing neuronal signals, such as the vasoactive intestinal peptide receptor 2 (VIPR2) [65, 66, 67, 68], neuromedin U receptor 1 (NMUR1) [69, 70, 71], beta‐adrenergic receptor 2 (β2AR) [72], dopamine receptors [73], calcitonin gene‐related peptide (CGRP) receptors [74, 75, 76], and various metabotropic acetylcholine receptors [77, 78]. Acetylcholine, NMU, and VIP stimulate ILC2s, whereas norepinephrine and dopamine inhibit ILC2 activation. CGRP has been shown to exert both activating and inhibitory effects on ILC2s, as it can induce IL‐5 and Areg while simultaneously suppressing IL‐13 production and limiting ILC2 proliferation. Thus, enteric neurons can directly regulate ILC2s, but recent evidence suggests that the enteric nervous system also influences the differentiation of epithelial stem cells into the secretory lineage, including tuft cells. Epithelial stem cells express VIPR1 and sense neuronal VIP. Deficiency in either VIP or VIPR1 leads to massive expansion of tuft cells, driving ILC2 activation via IL‐25 secretion [79, 80].

Finally, ILC2s express nuclear receptors, such as retinoic acid receptors (RAR) [28], aryl hydrocarbon receptor (AHR) [30], and androgen receptors [29], and their activation status is also determined by diet‐derived vitamin A and AHR ligands and by the sex hormone androgen.

### 1.2 ILC2 Effector Cytokines

ILC2s produce typical type 2 effector cytokines to drive type 2 immune responses. One of the main effector cytokines produced by ILC2s is IL‐5, which was originally described in T cells and termed “T cell‐replacing factor” due to its ability to initiate B cell differentiation in the absence of T cells [81, 82]. IL‐5 engages on the IL‐5 receptor detectable on murine B1‐cells and eosinophils [83]. In vivo gain‐of‐function experiments using IL‐5 transgenic mice already revealed systemic eosinophilia and the generation of polyreactive IgM antibodies in the early 1990s [84]. Conversely, mice deficient in IL‐5 or IL‐5Rα showed decreased numbers of B1‐cells and eosinophils, indicating the strict dependence on IL‐5 for the generation, activation, and survival of both cell types [85, 86]. While IL‐5 is linked to the pathogenesis of allergic inflammation, such as allergic asthma or eosinophilic esophagitis, the role during worm infections is limited to certain stages or models [87]. Instead, IL‐13 presents itself as a dominant effector cytokine for worm resistance by acting on different cell types, including epithelial cells and neurons [88, 89, 90, 91, 92]. IL‐13 engages on two cognate receptors, IL‐13rα1 and the decoy receptor IL‐13rα2. IL‐13rα1 is widely expressed in human and mouse immune and nonimmune cells except for T cells and murine B cells. Its activation leads to the initiation of the Janus tyrosine kinases (JAK)‐STAT6 signaling cascade and induces transcription of downstream genes. IL‐13 binds with low affinity to the IL‐13rα1, but when paired with IL‐4rα, a functional high‐affinity receptor complex, coined type II IL‐4 receptor, is formed, suggesting that IL‐13 shares many functional properties with IL‐4 [93]. However, IL‐13 is strictly required for worm clearance because *Il13* ^−/−^ mice are susceptible to *N. brasiliensis* infection [94, 95], whereas *Il4* ^−/−^ mice do not show delayed worm clearance [96]. IL‐13 has a broad array of important physiologic functions on hematopoietic cells, such as suppression of proinflammatory cytokine expression and upregulation of adhesion molecules in monocytes and macrophages [97]. In the nonhematopoietic compartment, IL‐13 has been linked to increased mucus production and airway hyperreactivity in the lung [98]. In the intestine, IL‐13 induces tuft and goblet cell hyperplasia, promoting mucus production for the “weep and sweep” reaction to expel the parasite [31, 99]. Interestingly, the *Il13* gene is located only around 13 kb apart from the gene encoding for *Il4*, both of which are coordinately regulated during type 2 immune responses [100]. Although IL‐4 can also be expressed by ILC2, it is mainly secreted by Th2 cells, and ILC2‐deficient mice have increased IL‐4 levels during worm infection [101]. IL‐4 engages on two receptor complexes: the type I receptor complex, expressed on hematopoietic cells, and the type II receptor complex found on many hematopoietic and nonhematopoietic cell types. Because IL‐13 similarly uses the type II receptor complex of the IL‐4 receptor, a certain overlap in effector functions exists. However, unlike IL‐13, IL‐4 engages the cognate receptor expression of complex I and is a potent inducer of IgE class‐switch recombination [102] and is involved in Th2 skewing [103]. While IL‐4 is essential for IgE switching in both mice and humans, IL‐13 can additionally promote IgE class switching in humans [104].

ILC2s also secrete the molecule amphiregulin (Areg), a ligand for the epidermal growth factor receptor. Areg was originally described as an essential effector molecule against *Trichuris muris* but not *N. brasiliensis* infection [105, 106], where it can stimulate epithelial shedding and T cell expansion. ILC2‐derived Areg has the ability to protect and repair epithelial tissue during damage caused by lung‐influenza infection or colitis [107, 108].

Taken together, the main effector cytokines expressed by ILC2s coordinate the type 2 immune machinery, supporting beneficial anti‐helminth immunity while also driving type 2 hyperactivated immune diseases, such as allergic asthma, in both humans and mice. Beyond these canonical effector functions, ILC2s have been connected with the production of the pleiotropic cytokine IL‐9 [57]. Originally, IL‐9 has been linked to mast cell activation [109], but later studies found that IL‐9 can act as an autocrine feed‐forward cytokine molecule, further boosting ILC2 activation and the consequential ILC2 response [57, 58]. However, the exact role of innate cell‐derived IL‐9 remains understudied. In addition to cytokine‐mediated immune regulation, ILC2s also mediate cellular regeneration within the bone marrow via the secretion of granulocyte‐macrophage colony‐stimulating factor (GM‐CSF). Under homeostatic conditions, the bone marrow of GM‐CSF knockout mice (*Csf2* ^−/−^) shows no major perturbances in cellular distribution. However, stressed bone marrow from GM‐CSF‐deficient mice following application of 5‐fluorouracil failed to recover the hematopoietic compartment. This effect was rescued via adoptive transfer of wild‐type ILC2s, but not GM‐CSF‐deficient ILC2s [110], suggesting that there are important ILC effector niches beyond mucosal tissues. Further, ILC2s act as major coordinators of immune cell migration and homing via expression of leukemia inhibitory factor (LIF). ILC2‐derived LIF promotes the production of CCL21 by the endothelium in the lung vasculature, orchestrating the egress of immune cells to mediastinal lymph nodes [111]. Taken together, ILC2s produce key signaling molecules that govern type 2 immune response, immune cell trafficking, as well as epithelial repair and regeneration.

### 1.3 Redundant versus Nonredundant Functions of ILC2s in Homeostasis and Type 2 Immunity

ILCs are innate lymphocytes with similar lineage‐specifying transcription factors and effector cytokines as their adaptive T cell counterparts but lacking rearranged antigen‐specific receptors. Intuitively, similarities between ILCs and T cells in effector functions could argue for overlapping functions and the redundant biological takeover in case of cellular loss of one immune cell population. However, the acquisition of the powerful rearranged antigen receptor capable of mediating memory immune response came at the price of tight control with multiple checkpoints, resulting in various consequences that might explain the different roles ILCs and T cells play during an immune response. From a descriptive standpoint, the kinetics of the response, regulation of effector functions, and spatial distribution within tissues differ between ILCs and T cells.

Differences in effector cytokine production are highlighted by the finding that ILCs already play an important role in tissue homeostasis during development, a stage when antigen‐specific T cells have barely entered the tissue [112]. At the whole‐organ level, the most notable example is the function of lymphoid tissue inducer cells (LTi cells), an ILC3 subset essential in the formation of secondary lymphoid organ development, as evidenced by the lack of the vast majority of lymph nodes in mice deficient in LTi cells [17, 113]. Consistently, the absence of palpable axillary and cervical lymph nodes was reported from humans with a deficiency in the lineage specifying transcription factor RORc [114]. Therefore, ILC3s have essential functions in the development of secondary lymph organs in mice and humans.

In addition, published data argue for a strict requirement of ILCs to produce essential factors for the development of immune cell types. As a distinguishing feature, several ILC subsets, including ILC2, are characterized by the tonic secretion of effector cytokines and epigenetic marks at poised cytokine loci before activation. In contrast, T cells require prior activation by antigen‐presenting cells to acquire a similar signature. Th2 cells acquire similar epigenetic marks, for example, at the *Il5* and *Il13* locus following worm infection [115]. However, an important consideration is that much of this work has been performed in germ‐free or SPF mouse models, where Th2 cells lack the chronic environmental stimulation and microbial exposures characteristic of humans. It is therefore plausible that, in humans continually shaped by commensal microbiota, repeated allergen exposure, and tissue‐specific inflammatory histories, Th2 cells may exhibit a more “pre‐poised” or partially primed epigenetic landscape that more closely resembles that of ILC2s. Whether human Th2 and ILC2 populations converge epigenetically under such conditions remains largely unexplored and represents an important area for future investigations. As a consequence of their constitutive epigenetic accessibility and independence from antigen priming, ILC2s remain the dominant source of IL‐5 in mucosal tissues at steady state [68].

Several mouse models were developed with the aim of targeting ILC2 while sparing Th2 cells. Although GATA‐3 is expressed and developmentally required for ILC2 and Th2 cells, some enhancer elements mainly support expression in one cell type. For instance, deletion of the enhancer region *Gata3* +674/762 affected ILC2 while only minimally impairing Th2 cell differentiation [116]. Furthermore, an ILC2‐specific locus control region within the *Id2* promoter was identified that is required for three‐dimensional genome organization. Deletion of this locus control region impaired ILC2 development but did not affect Th2 cells [117]. In addition, several Cre‐driver mouse lines have been developed to restrict transcription factor deletion to ILC2s or enable their genetic ablation via expression of a diphtheria toxin cassette, such as *the Nmur1* ^Cre^ *Id2* ^fl/fl^ [101, 118] mouse line, the *Klrg1* ^Cre^ *Gata3* ^fl/fl^ [119], or *Il5* ^Cre^ DTA lines [68].

Taken together, these mouse models have provided compelling evidence for nonredundant functions of ILC2s at steady state and during infection or inflammation. For example, various mouse models demonstrate that interference with ILC2s, ILC2‐derived IL‐5 secretion, or interference with the upstream IL‐33 pathways strongly impairs the development and function of eosinophils and B1 cells [46, 68, 120, 121]. In contrast, deletion of the cytokine IL‐5 in T cells did not affect B1 cell development, supporting the notion that ILC2s are the dominant source of IL‐5, whereas the contribution of T cells is negligible at steady state [120]. As a consequence of the altered ILC2‐dependent tissue homeostasis, eosinophilia was diminished in the context of type 2 immunity, for example, during allergic lung inflammation or worm infection [101, 118, 119, 122]. Moreover, the importance of the ILC2‐derived cytokines IL‐13 and Areg becomes evident during worm infection and inflammation. As mentioned earlier, IL‐13 targets epithelial cells and induces tuft and goblet cell hyperplasia and thus represents the decisive cytokine triggering the weep‐and‐sweep reaction to expel the parasite [13, 14, 101, 118, 123]. Consequently, genetic interference with ILC2s or their effector cytokines results in impaired tuft and goblet cell hyperplasia and increased susceptibility to worm infection, defects that cannot be compensated by T cells in the acute phase, indicating that ILCs are nonredundant, pivotal players during health and disease [31, 101, 118, 119, 122].

Taken together, the requirement for ILC2s for acute worm infections, especially during *N. brasiliensis* infection is now well established and based on the usage of several genetic models to interfere with ILC2s and avoid depleting Th2 cells as the adaptive counterpart [124]. More importantly, the contribution of ILC subsets in covering the early phase of acute infections appears to be a common characteristic of several ILC subsets. Ivanov's group recently generated mice that selectively lack ILC3 but contain Th17 cells to investigate the role of ILC3s in protective immunity against enteropathogenic *Escherichia coli* strain *Citrobacter rodentium* in lymphoreplete mice. Earlier work has established the importance of IL‐22 for resistance against *C. rodentium* and identified ILC3s as the main IL‐22‐producing cell type in the acute phase of infection [125, 126]. However, whether lack of ILC3s could be compensated by Th17 was not clarified. Mice with selective depletion of ILC3s succumbed to high‐dose *C. rodentium*, providing evidence for an essential function of ILC3s in acute *C. rodentium* infection [127]. In contrast, ILC3s were dispensable for the control of chronic *C. rodentium* infection. Thus, this study complements numerous reports assigning an important function for ILC3‐derived IL‐22 in fighting acute infections, containment of bacteria, and maintenance of the epithelial barrier integrity [128, 129, 130, 131, 132]. Collectively, the data obtained in models of selective ILC2 and ILC3‐deletion suggest a pivotal role for ILCs in acute infections, which cannot be compensated for by the adaptive T‐cell counterparts.

Moreover, evidence from decades of NK cell research has established NK cells as a pivotal cell type to fight cytomegalovirus (CMV) infection in mice and humans. This includes multiple mouse strains with impaired NK cell function or a complete lack of NK cells, correlating with resilience against murine CMV infection. In humans, patients with GATA‐2 deficiency were reported to be susceptible to HCMV infection along with a defect in NK cell function [133, 134], clearly underlining the role played by ILCs in host physiology also in humans. Finally, from the perspective of the pathogen, the herpes virus produces a myriad of molecules that interfere with NK cell recognition and NKG2D ligands or MHC I molecules, highlighting the investment of the pathogen to escape NK cell immunosurveillance [135].

A potentially redundant role of ILCs has been suggested by studies of patients with severe combined immunodeficiency (SCID) who underwent hematopoietic stem cell transplantation (HSCT). After HSCT, successful T cell reconstitution was obtained, whereas efficient reconstitution of the ILC compartment was not observed. Nevertheless, these patients survived and did not develop severe diseases evident in long‐term follow‐up [136]. However, nearly half of patients with innate lymphocyte deficiency tested positive for human papillomavirus and had additional infections and sequelae related to barrier immunity [137]. Moreover, a follow‐up study in SCID patients focusing on the mucosal immune compartment found defects in type 2 cytokines and immune cells, including ILC2s, as well as T cell subsets, along with differences in IgA levels, mucus production, and the microbiota [138]. Therefore, these findings highlight the need for additional studies to investigate the precise contributions of ILCs and T cell subsets in fighting infections and maintaining tissue homeostasis in humans.

ILCs are abundant in many human tissues, particularly the intestine, but are also detectable in the peripheral blood, raising the question of whether T cells can fully compensate for ILCs [139]. In mice, in contrast to T cells, ILCs are considered tissue‐resident cells, and their ability to patrol and circulate to tissues is limited. This finding has been studied in mice surgically joined to establish blood chimerism, where T and B cells equilibrated between partners, whereas most ILCs did not [24]. However, during type 2 inflammation, iILC2s can acquire migratory features, for example, from the small intestine to the lung, to augment type 2 immune responses [7, 27, 63, 140]. Conversely, naive T cells are constantly replenished in tissues and require migration to secondary lymphoid organs for their activation and TCR selection. These differences in migration capacity and tissue behavior comparing ILCs and T cells may suggest that the innate immune system maintains tissue homeostasis at steady state through bystander cytokine secretion and by priming the local milieu for rapid immune responses, whereas the adaptive T cell compartment optimizes immune response by generating antigen‐specific memory through permanent circulation and renewal. However, it is also worth noting that tissue‐resident memory (TRMs) T cells share key features with ILCs. Although traditionally viewed as antigen‐dependent, accumulating evidence suggests that Th2 TRM cells can persist long‐term in barrier tissues, such as the lung, gut, or skin, and may contribute to low‐level, homeostatic type 2 cytokine production often occupying similar niches near epithelial barriers [141]. In addition to Th2 TRM cells, several unconventional T cell subsets, including type‐2–polarized CD8^+^ T cells, invariant NKT (iNKT) cells, and γδ T cells, are capable of producing IL‐4, IL‐5, and IL‐13 under certain conditions [142]. These populations may therefore complement or partially overlap with ILC2 functions in maintaining tissue tone, initiating early type 2 responses, or shaping the cytokine milieu before full adaptive activation. Understanding how these resident and innate‐like T cell compartments integrate with, or compete against ILC2s, remains an important unresolved question with significant implications for barrier immunity and chronic type 2 inflammation. In summary, these characteristics allow both cell types to provide rapid, localized immune responses while maintaining long‐term presence in their respective tissues.

Accumulating evidence supports a critical role for ILC2s in shaping adaptive Th2‐cell responses. ILC2‐derived IL‐13 promotes DC activation and lymph node migration, enabling naïve CD4^+^ T cells to differentiate into Th2 cells even in the absence of IL‐4 [143]. Moreover, ILC2s expressing MHC class II can directly dialog with antigen‐specific T cells, supporting mutual expansion and cytokine production during parasite infection [144]. Finally, ILC2s are required for efficient recruitment and reactivation of memory Th2 cells upon allergen re‐challenge [145]. Together, these studies highlight that ILC2s are not merely an innate parallel of Th2 cells, but active orchestrators and potentiators of adaptive type 2 immunity.

Thus, ILCs and T cells complement each other to induce an optimal immune response. Overall, despite the substantial similarities between ILCs and their adaptive T cell counterparts, accumulating evidence clearly indicates the unique and nonredundant functions of ILCs in host physiology, which cannot be fully compensated by T cells.

### 1.4 Targeting Alarmins and Type 2 Effector Cytokines in Human Disease

Even though type 2 immune responses are beneficial for fighting against parasitic infections and maintaining the delicate equilibrium in tissues, they become hyperactivated in the context of allergic asthma, atopic dermatitis, eosinophilic esophagitis, and food allergies. Each step of the type 2 immune cascade has lately evolved as a targeting strategy to precisely treat affected patients [146, 147]. Monoclonal antibodies targeting certain branches of the type 2 immune response have become standard of care or are currently being tested in clinical trials. Among these, inhibition of the alarmin IL‐33 via Itepekimab or Tozorakimab is being tested for the treatment of asthma and chronic obstructive pulmonary disease [148]. In addition, Tezepelumab [149] neutralizes the alarmin TSLP and is approved for the treatment of severe asthma [150]. Furthermore, antagonism of the ILC2 effector cytokines IL‐5 (Mepolizumab, Reslizumab) and IL‐13 (Lebrikizumab, Tralokinumab) or components of the corresponding receptors IL‐5Rα (Benralizumab) and IL‐4Rα (Dupilumab) have evolved as milestones in the treatment of type 2 immune diseases [151]. Based on available therapeutic options, precision medicine approaches enable selective inhibition of certain hyperactivated immune sequelae during disease and allow treatment to be customized to individual patient profiles. Although these interventions are not specific for ILC2s, they directly interfere with main up‐ and downstream molecules required for ILC2 activation or effector functions. The plethora of cytokines related to ILC2 biology that have successfully been targeted by biologics strongly suggests a contributory role for ILC2s in disease progression. However, further investigations are necessary to dissect ILC2‐specific effects. Given the broad array of functions where ILC2s are critically involved, inhibition/stimulation of the type 2 immune cascade may be a further valuable option for treating fibrosis, cancer, or adiposity. Future research focusing on molecular mechanisms regulating ILC2 activation, plasticity, migration, and interactions with adaptive immune cells and the nervous system is likely to reveal novel strategies for modulating and selectively targeting chronic type 2 inflammation, while preserving protective immune functions.

## Author Contributions

M.O.J. and C.S.N.K. wrote the manuscript together. P.M.F critically reviewed the manuscript and designed the illustrations.

## Conflicts of Interest

The authors declare no conflicts of interest.

## Data Availability

Data sharing is not applicable to this article as no datasets were generated or analyzed during the current study.
